# Dispatcher Self-assessment and Attitude Toward Video Assistance as a New Tool in Simulated Cardiopulmonary Resuscitation

**DOI:** 10.5811/westjem.2021.12.53027

**Published:** 2022-02-28

**Authors:** Hannes Ecker, Sabine Wingen, Anna Hagemeier, Christopher Plata, Bernd W. Böttiger, Wolfgang A. Wetsch

**Affiliations:** * University of Cologne, Medical Faculty and University Hospital Cologne, Department of Anaesthesiology and Intensive Care Medicine, Cologne, Germany; † FOM University of Applied Sciences, Cologne, Germany; ‡ University of Cologne, Medical Faculty and University Hospital Cologne, Institute of Medical Statistics and Computational Biology, Cologne, Germany; § University Hospital RWTH Aachen, Department of Emergency Medicine, Aachen, Germany

## Abstract

**Introduction:**

Video-assisted cardiopulmonary resuscitation (V-CPR) describes an advanced telephone-assisted CPR (T-CPR), in which emergency medical service (EMS) dispatchers view a live video steam of the resuscitation. Dispatchers ‘ general attitudes toward and self-assessment in V-CPR have not been previously investigated.

**Material and Methods:**

We conducted this quantitative analysis along with a pilot study on V-CPR. After conducting V-CPR with laypersons in a simulation, EMS dispatchers were given questionnaires with 21 items concerning their personal attitude toward V-CPR and their self-assessment in providing instructions. The actual CPR performance achieved was recorded and compared to the dispatchers’ self-assessments.

**Results:**

Dispatchers completed 49 questionnaires, and the data is presented descriptively. Over 80% strongly agreed that V-CPR was helpful in guiding and that their feedback improved CPR quality. Fifty-one percent agreed that video images supported them in making a diagnosis, while 44.9% disagreed. A vast majority (80–90% each) strongly agreed that V-CPR helped them recognize CPR issues such as compression point, compression rate, and deterioration. In contrast, data for improved compression depth and release were weaker. Thirty percent found V-CPR to be more stressful or exhausting than T-CPR. A majority stated they would prefer V-CPR as an addition to T-CPR in the future. There was a huge gap between dispatchers’ own view of CPR effort and measured CPR quality.

**Conclusion:**

Dispatchers generally embrace V-CPR and praise the abilities it provides. Our results indicate that the use of V-CPR did not automatically result in an overall improvement in guideline-compliant CPR quality.

## INTRODUCTION

Telephone assistance by emergency medical service (EMS) dispatchers during cardiopulmonary resuscitation (CPR) has proven to improve patient outcome and is strongly endorsed by international guidelines.^1^ Dispatchers are trained in providing telephone-assisted CPR (T-CPR) when cardiac arrest is suspected or recognized. The dispatcher then follows a structured protocol and gives the caller systematic instructions on how to perform CPR. However, this widely implemented routine is limited by several factors. For example, the callers’ depiction of the situation may not be accurate due to personal limitations as well as to the potentially stressful emergency situation. Even if the dispatchers’ instructions are presumed to be followed, they remain unaware of whether the CPR is being performed correctly or sufficiently.

To overcome this uncertainty and improve patient outcome in cardiac arrest, novel technologies now enable the dispatcher to see a live video stream from the caller’s smartphone of the actual CPR effort. This facilitates video-assisted CPR (V-CPR). Research on the effectiveness of V-CPR has been focused primarily on resuscitation quality and recognition of typical mistakes.^2^ However, studies have yet to evaluate EMS dispatchers’ attitudes about this technology.^3^–^9^ In this study, we evaluated the dispatchers’ attitudes toward V-CPR using a post-interventional questionnaire after a simulated V-CPR situation, to get their quantitative assessment of this technology.

## METHODS

### Ethics Approval

The Ethics Committee of the University of Cologne approved the study (ID 18-043; 2018-03-27; Head: Prof. Dr. Drzezga), which was conducted in accordance with CONSORT guidelines specifically under the pilot and feasibility statement.

### Study Registration

The study was registered at ClinicalTrials.gov (Identifier: NCT03527771).

### Study Design

This quantitative analysis was conducted in a subgroup of a pilot study on V-CPR from July–August 2018 in the facilities of the University Hospital of Cologne.^7^ In our skills lab, we set up a full-scale CPR manikin (Ambu Man Advanced, Ambu A/S, Ballerup, Denmark) to simulate a patient in cardiac arrest. In a second room, an emergency dispatcher workplace was set up, including a telephone, a computer with emergency dispatcher software (iSE COBRA 4, iSE GmbH, Aachen, Germany), and four displays with full high-definition 16:9 capability. In addition, a novel software (EmergencyEye, Corevas GmbH & Co. KG, Grevenbroich, Germany) was installed on the dispatching computer, which enables a one-way video telephone connection to a smartphone. The dispatchers consisted of four experienced emergency medical dispatchers in rotation who averaged greater than 10 years of dispatching experience, and all were trained paramedics with experience in the field. There was no randomization for the dispatcher rotation, as available personnel were used according to the duty roster of the dispatchers’ employer.

Fifty healthy adult volunteers with no medical background were recruited from passersby at the study location. Volunteers were blinded to the study objective. They were told that they would be confronted with a medical emergency situation and were instructed to handle it to the best of their ability. They were given a study smartphone to “call EMS if necessary.” After giving written and informed consent, these participants were accompanied into the simulation room by a study assistant who was equipped with a smartphone (Samsung S7, Android 7.1.1, Samsung Group, Seoul, Korea). Participants were instructed to provide “appropriate first aid according to their own best knowledge and skills.” During the CPR scenario, using the phone for an emergency call automatically established a video connection from the caller’s phone to the dispatch center. The study assistant operated the phone and placed himself with the camera facing participants and manikin in a predefined position.

**Population Health Research Capsule**

What do we already know about this issue?

*Telephone assistance by Emergency Medical Services (EMS) dispatchers during CPR improves patient outcomes. Video-assisted CPR (V-CPR) could be a consistent further development of the current Telephone-CPR (T-CPR).*

What was the research question?

*We evaluated dispatchers’ attitude toward V-CPR using a post–interventional questionnaire after a simulated V-CPR situation.*

What was the major finding of the study?

*Although dispatchers embrace the abilities of V-CPR, there is a discrepancy between the assessment and actual quality of CPR.*

How does this improve population health?

*While V-CPR has the potential to strongly improve resuscitation quality, it is important to train and guide EMS dispatchers in V-CPR to sustain the best results.*

The dispatcher followed a standardized, structured dispatching protocol, quickly guiding the caller through the most important questions and toward the correct diagnosis. After detection of cardiac arrest, the dispatcher informed the participants that the patient was in cardiac arrest and needed resuscitation. The dispatcher then activated the video livestream and started standardized, video-guided compression-only CPR according to the 2015 European Resuscitation Council Guidelines for Resuscitation.^1^ Resuscitation quality was recorded via the integrated software of the CPR manikin (see above) and analyzed afterward. All scenarios were to be terminated after eight minutes of CPR.

After each V-CPR, the dispatcher was given a questionnaire we developed, which consisted of 21 items focusing on the dispatcher’s personal attitude toward V-CPR and its usefulness in providing CPR instructions. For each question, answers could be given on a four-point Likert scale. These questionnaires were then descriptively and quantitatively analyzed. We conducted descriptive analysis using SPSS version 25 (IBM Corp., Armonk, NY). Results are presented as absolute and relative frequencies.

## RESULTS

Fifty V-CPR sequences were eligible to be analyzed in this study. In one case, due to technical problems no video was transmitted, and hence no questionnaire could be completed. All remaining 49 attempts were successful and resulted in instructed V-CPR for eight minutes. In all these cases, a questionnaire was completed by the dispatcher in charge. Thus, 49 complete questionnaires were obtained for further analysis.

### Average Age, Gender and Experience in EMS Dispatching and Telephone-CPR

In this study the mean age of the dispatchers was 54 years ± 6.9 years. All participating dispatchers were male, and all had more than 10 years experience as EMS dispatchers.

### Evaluation of General Usefulness of Video-CPR (Items 1–4)

When asked whether the video image irritated them while instructing CPR, 95.9% of the dispatchers strongly disagreed (Item 1). Over 80% strongly agreed that V-CPR was helpful in guiding subjects in CPR (Item 2) and 75.5% strongly agreed, while the rest agreed that the video image offered a new quality in the emergency call inquiry (Item 3). Regarding the question of whether the video image helped with the emergency call inquiry, only 55.1% strongly agreed and 38.8% agreed, while 6.1% disagreed (Item 4).

### Evaluation of Recognizing Certain CPR Issues (Items 5–12)

Fifty-one percent agreed that video streaming supported them in making a diagnosis, while 44.9% disagreed with that statement (Item 5). In contrast, 85.7% strongly agreed that the video showed them errors in the resuscitation effort of the subject (Item 6), while 14.3% disagreed. Regarding certain CPR issues, such as correct compression point (Items 7), rate (Item 8) and compression fatigue (Item 11), 81.6%, 93.9% and 81.6%, respectively, strongly agreed that V-CPR helped them to recognize mistakes. For CPR quality issues, such as compression depth and release (Items 9 and 10), only 59.2% and 63.3% strongly agreed, while 18.4% and 20.4 % disagreed. Still, 83% strongly agreed that they were able to see that their feedback improved the quality of the CPR effort through the video stream, while only 2% disagreed (Item 12).

### Comparing V-CPR to T-CPR and General Acceptance (Item 13–21)

Over 77% of the EMS dispatchers found the image quality of the video to be sufficient, whereas 12% disagreed (Item 21). A majority of 95.9 % strongly agreed that V-CPR helped them recognize and give corrective feedback during CPR, which they would not have recognized with T-CPR only (Item 13). Additionally, 42% strongly agreed and 46% agreed that they had the impression V-CPR helped them motivate the subject more that T-CPR alone could have done (Item 14).

Interestingly, 30% agreed that they found the video-assisted method more stressful or exhausting than the telephone-assisted method, while 63% strongly disagreed (Item 20). Still, 75% strongly agreed and 24% agreed that V-CPR was helpful in guiding the subjects on CPR and resulted in better CPR quality (Item 15). In addition, 85% strongly agreed that video-assisted CPR facilitates the instruction of CPR (Item 16). Regarding their own bias, 28% admitted that they were skeptical about video technology used in CPR, while 38% and 30%, respectively, disagreed or strongly disagreed that they were skeptical before (Item 19).

Eighty-three percent disagreed that they would prefer T-CPR only in the future (Item 17). Sixty-one percent strongly agreed and 38% agreed that that they would like to see V-CPR as an aid in their dispatching work in the future (Item 18). A summary of the questionnaire and the corresponding results is shown in Table 1.

### Comparison of Dispatchers’ Perceptions and Measured Values of Compression Rate and Depth

**Figure 1:**
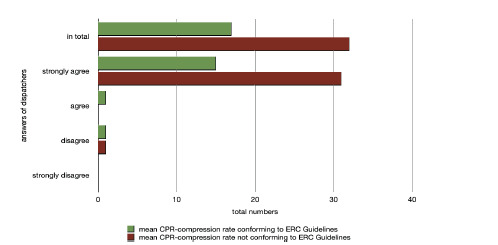
Agreement with Item 8 “The video image let me see errors in thorax compression rate” in comparison to mean CPR compression depth achieved. *CPR,* cardiopulmonary resuscitation; *ERC,* European Resuscitation Council.

As actual CPR data about compression rate and depth was stored via the onboard software of the manikin, a direct comparison to the assessment of the dispatchers was possible. Regarding the statement of item 8, “The video image let me see errors in thorax compression rate,” 93.9% strongly agreed. Comparing it to the actual compression rate achieved in these cases, it became obvious that only 32.6% performed a correct mean compression rate over eight minutes, while the majority (67.4%) did not perform a correct mean compression rate recommended by the international guidelines on resuscitation (Figure 1).

**Figure 2:**
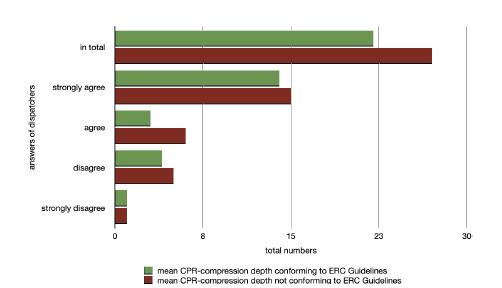
Agreement with Item 9 “The video image let me see errors in thorax compression depth” in comparison to mean CPR compression depth achieved. *CPR,* cardiopulmonary resuscitation; *ERC,* European Resuscitation Council.

Regarding item 9, “The video image let me see errors in thorax compression depth,” approximately 60% strongly agreed that only 48.3% of the volunteers actually performed a mean compression depth over eight minutes according to the guidelines (Figure 2).

## DISCUSSION

Video-assisted CPR is a new tool for dispatchers to guide callers in providing CPR. While some studies focusing on the impact of V-CPR on CPR quality have been conducted, there is a paucity of research regarding EMS dispatchers’ attitudes to V-CPR.^2^ We undertook this study to investigate in a quantitative fashion the dispatchers’ acceptance of and attitudes toward V-CPR.

While the concept of V-CPR has been around for over a decade, its large-scale deployment in EMS was never achieved due to technical limitations.^2^,^3^ However, recent progress in smartphone technology and broad bandwidth internet coverage has made its utilization possible in emergencies.^8^,^9^ Several studies have shown its advantages and superiority in comparison to T-CPR, despite the sometimes ambivalent results.^3^,^9^

A similar study to ours was conducted in 2008 in Norway. There, dispatchers were interviewed after simulated V-CPR sessions (with a Nokia N90). The answers in those taped interviews were then coded into categories, analyzed, and descriptively presented. The authors concluded that the video element improved the dispatchers’ understanding of the emergency situation and that it assisted them in their work. But in their assessment, the dispatchers also stated that the video picture could “interfere” with their normal protocol, as it sometimes could be “noisy,” “chaotic,” and distracting. They also complained that the image quality was sometimes insufficient, as motion of the camera led to “interference.”^3^

With our study, conducted over a decade later with state-of-the-art technology, we tried to systematically investigate V-CPR acceptance and usefulness from the perspectives of EMS dispatchers, who are the key players in its utilization. The overwhelming majority of the dispatchers in our study felt that V-CPR was beneficial to their work, helpful in instructing CPR, and offered a new quality in the emergency query process. However, dispatchers were nearly split in their attitude of V-CPR helping them with their diagnosis, with a trend toward agreement.

Dispatchers in general strongly agreed that the video stream showed them errors in the resuscitation effort. Namely, dispatchers in 80–90% of the cases strongly agreed it to be helpful in the recognition of the correct compression point, frequency, and compression fatigue. In contrast, only around 60% strongly agreed that V-CPR helped them to recognize mistakes in compression depth and release. Interestingly, however, we found a huge discrepancy between the assessment of the observed CPR and the actual performed CPR – as compression rate and depth were only correctly performed in 32% and 48% of the cases, respectively, in which the dispatchers strongly agreed that V-CPR helped them in recognizing these mistakes.

The reason for this surprising discrepancy is not clear. On the one hand, this might be attributed to technical reasons, such as image quality, video frame rate, or the angle in which the scene was being filmed. Contrary to this, most dispatchers found that the video quality was sufficient to evaluate CPR, which is in strong contrast to studies from over a decade ago, in which V-CPR was deemed unfavorable due to its poor video quality.^4^ Technical evolution over a decade may have had significant impact on video quality and may pose an explanation as to why this finding changed over time.

Another important reason for this contradiction might be attributed to the dispatchers’ operating experience with V-CPR. Even though trained and seasoned in T-CPR, the video element represents a new aspect to their work, as dispatchers are not yet accustomed to visually evaluate a live CPR. This might be enforced by our finding that about 30% conceded and agreed that the video-assisted method was more stressful or exhausting than normal T-CPR (while 63% strongly disagreed). We conclude that these might be indicators that dispatchers need special theoretical-practical training and perhaps emotional support before using V-CPR. Still, the majority of dispatchers in our study agreed that V-CPR facilitated their task and was advantageous to T-CPR and reported that they would like to see V-CPR become part of their dispatching work in the future.

## LIMITATIONS

This study investigated a small group of only male, older, and very experienced dispatchers in an artificial scenario on a CPR manikin. Further studies should be conducted on a larger scale and perhaps in a real emergency situation with all genders to increase its generalizability.

## CONCLUSION

Video-assisted CPR is a novel technology that was highly embraced by EMS dispatchers in our study. Dispatchers praised the new abilities provided by it. However, our results indicate that the benefits of V-CPR are not a given, as use of V-CPR did not automatically result in an overall improvement in guideline-compliant CPR quality. Dispatchers need further training and guidance to integrate V-CPR into their workflow.

## Figures and Tables

**Table 1:** Distribution of dispatchers’ agreement/disagreement.

	Strongly agree	Agree	Disagree	Strongly disagree
Item 1: The video image irritated me when I was instructing the subjects on CPR.	0 (0.0%)	0 (0.0%)	2 (4.1%)	47 (95.9%)
Item 2: Video-assisted CPR was helpful in guiding subjects in CPR.	40 (81.6%)	9 (18.4%)	0 (0.0%)	0 (0.0%)
Item 3: The video image offers a new quality in the emergency call inquiry.	37 (75.5%)	12 (24.5%)	0 (0.0%)	0 (0.0%)
Item 4: The video image helped me with the emergency call inquiry.	27 (55.1%)	19 (38.8%)	3 (6.1%)	0 (0.0%)
Item 5: The video image supported me in making a diagnosis.	2 (4.1%)	25 (51.0%)	22 (44.9%)	0 (0.0%)
Item 6: The video showed me errors in the resuscitation effort of the subject.	42 (85.7%)	7 (14.3%)	0 (0.0%)	0 (0.0%)
Item 7: The video image let me see errors of the correct thorax compression point.	40 (81.6%)	6 (12.2%)	2 (4.1%)	1 (2.0%)
Item 8: The video image let me see errors in thorax compression rate.	46 (93.9%)	1 (2.0%)	2 (4.1%)	0 (0.0%)
Item 9: The video image let me see errors in thorax compression depth.	29 (59.2%)	9 (18.4%)	9 (18.4%)	2 (4.1%)
Item 10: The video image let me see errors in the thorax compression release.	31 (63.3%)	5 (10.2%)	10 (20.4%)	3 (6.1%)
Item 11: Through the video image I was able to see signs of fatigue / deteriorating quality in the resuscitation effort.	40 (81.6%)	7 (14.3%)	2 (4.1%)	0 (0.0%)
Item 12: Through the video stream I was able to see that my feedback helped improving the quality of the resuscitation effort.	41 (83.7%)	7 (14.3%)	1 (2.0%)	0 (0.0%)
Item 13: Through the video-assisted CPR I was able to recognize and correct errors in the CPR, which I would not have recognized with telephone-assisted CPR only.	47 (95.9%)	2 (4.1%)	0 (0.0%)	0 (0.0%)
Item 14: I had the impression that I was able to motivate the test subject better with video-assisted CPR than I could have done with telephone-assisted CPR alone.	21 (42.9%)	23 (46.9%)	5 (10.2%)	0 (0.0%)
Item 15: Video-assisted CPR was helpful in guiding the subjects on CPR and resulted in better CPR quality.	37 (75.5%)	12 (24.5%)	0 (0.0%)	0 (0.0%)
Item 16: The video-assisted CPR facilitates the instruction of the subjects for CPR.	42 (85.7%)	7 (14.3%)	0 (0.0%)	0 (0.0%)
Item 17: I still prefer telephone CPR alone in the future.	0 (0.0%)	3 (6.1%)	5 (10.2%)	41 (83.7%)
Item 18: I would like to see video-assisted CPR as an aid in my work as a dispatcher in the future.	30 (61.2%)	19 (38.8%)	0 (0.0%)	0 (0.0%)
Item 19: Were you skeptical about the video technology used in CPR?	1 (2.0%)	14 (28.6%)	19 (38.8%)	15 (30.6%)
Item 20: Did you find the video-assisted method more stressful or exhausting than the telephone-assisted method?	15 (30.6%)	1 (2.0%)	2 (4.1%)	31 (63.3%)
Item 21: Did you find the image quality of the video to be sufficient?	35 (71.4%)	8 (16.3%)	6 (12.2%)	0 (0.0%)
	< 1 year	1–5 years	5–10 years	>10 years
Item 22: Prior experience as EMS dispatchers	0 (0.0%)	0 (0.0%)	0 (0.0%)	49 (100%)

Questionnaire used for the study, with corresponding answers. Items 0–21 with the distribution of agreement – disagreement presented in absolute and relative frequencies (%).

*CPR*, cardiopulmonary resuscitation; *EMS*, emergency medical services.
